# Pulmonary impairment after tuberculosis in a South African population

**DOI:** 10.4102/sajp.v72i1.307

**Published:** 2016-06-30

**Authors:** Gibwa Cole, Duncan Miller, Tasneem Ebrahim, Tannith Dreyden, Rory Simpson, Shamila Manie

**Affiliations:** 1 Department of Physiotherapy, University of Cape Town, South Africa

## Abstract

**Background:**

In South Africa, pulmonary tuberculosis (PTB) remains a problem of epidemic proportions. Despite evidence demonstrating persistent lung impairment after PTB cure, few population-based South African studies have investigated this finding. Pulmonary rehabilitation post-cure is not routinely received.

**Objectives:**

To determine the effects of PTB on lung function in adults with current or past PTB. To determine any association between PTB and chronic obstructive pulmonary disease (COPD).

**Methods:**

This study was observational and cross-sectional in design. Participants (*n* = 55) were included if they were HIV positive on treatment, had current PTB and were on treatment, and/or had previous PTB and completed treatment or if they were healthy adult subjects with no history of PTB. A sample of convenience was used with participants coming from a similar socio-economic background and undergoing spirometry testing. Multiple regression analyses were conducted on each lung function variable.

**Results:**

Compared to normal percentage-predicted values, forced expiratory volume in 1 second (FEV_1_), forced vital capacity (FVC) and FEV_1_:FVC were significantly reduced in those with current PTB by 23.39%, 15.99% and 6.4%, respectively. Both FEV_1_ and FVC were significantly reduced in those with past PTB by 11.76% and 10.79%, respectively. There was no association between PTB and COPD – those with previous PTB having a reduced FEV_1_:FVC (4.88% less than the norm), which was just short of significance (*p* = 0.059).

**Conclusions:**

Lung function is reduced both during and after treatment for PTB and these deficits may persist. This has implications regarding the need for pulmonary rehabilitation even after medical cure.

## Introduction

Tuberculosis (TB) is a major global health problem and is ranked as the second leading cause of death from an infectious disease (World Health Organisation, i.e. WHO 2014).

South Africa is in the throes of a TB epidemic, having the third largest TB burden in the world after India and China (South Africa Department of Health 2014). Annual incidence rates have been reported to be 348 per 100 000 (smear-positive TB) and 948 per 100 000 (all types of TB) - these being the highest reported incidence rates in the world (Ehrlich *et al*. 2011a).

There is an emerging body of research detailing the residual effects of pulmonary TB (PTB) on lung function, namely a decrease in lung function parameters and contribution to the emergence of chronic obstructive pulmonary disease (COPD) (Akkara *et al*. 2013; Allwood, Myer & Bateman 2013; Maguire *et al*. 2009; Pasipanodya *et al*. 2007). In a review of South African studies specifically, Ehrlich *et al*. (2011a) found that there was a strong evidence to support the relationship between airflow obstruction and PTB. This means that a large number of people infected with PTB will continue to experience impairments in lung function even after treatment.

However, the majority of studies included in the review by Ehrlich *et al*. (2011a) were conducted on mining populations where adverse working conditions may have contributed to lung disease (Ross & Murray 2004). There is a marked lack of South African literature looking at PTB and COPD in community populations as the majority have focused on miners and industrial workers (Ehrlich *et al*. 2011b; Hessel & Sluis-Cremer 1989; Hnizdo 1992; Hnizdo, Singh & Churchyard 2000; Naidoo *et al*. 2005).

TB in South Africa is also fuelled by the concurrent HIV epidemic (South Africa Department of Health 2014) – those with HIV having an increased likelihood of contracting pulmonary infections and developing COPD (Crothers *et al*. 2011). Although research has shown greater declines in lung function with an increased viral load (Drummond *et al*. 2013), the precise contribution of HIV to PTB infection is yet to be determined. Despite the introduction of Highly Active Antiretroviral Therapy (HAART), PTB infection rates have remained high (Benito *et al*. 2012).

There is a dearth of research on physiotherapy interventions for PTB. Traditionally, patients with PTB do not receive physiotherapy except in cases where excessive secretions are problematic (Goodman & Fuller 2009). Additionally, patients do not routinely receive pulmonary rehabilitation after PTB cure despite research demonstrating its benefits (Ando *et al*. 2003; de Grass, Manie & Amosun 2014).

Spirometry is the tool most often used to assess lung function, and it measures volume against time (Ranu, Wilde & Madden 2011). Measurements include the following: (1) percentage-predicted forced vital capacity (FVC), which is the amount of air a person can forcefully expire after maximal inspiration; (2) percentage-predicted forced expiratory volume in 1 second (FEV_1_), the amount of air a person can forcefully expire in 1 second; and FEV_1_:FVC. These values are useful in identifying obstructive/restrictive respiratory deficiencies. If FEV_1_ is reduced more than FVC (FEV_1_:FVC is less than 70% of the predicted norm), this indicates an obstruction to airflow, for example, COPD. If FVC is reduced more than FEV_1_ (FEV_1_:FVC is more than 70% of the predicted norm), it signifies restricted volume, for example, chest wall deformities.

The aim of this study was to investigate the effects of PTB on lung function in a community population of South African adults. This study could add to the body of knowledge informing the need for physiotherapy post-PTB cure.

The main objectives were to determine the effects of PTB (current or past) on FEV_1_, FVC and FEV_1_:FVC and to determine any association between COPD and history of PTB, as outlined by GOLD standards for COPD (Dewar & Curry 2006).

## Research design

### Research method

#### Design

This study was observational and cross-sectional in design and used a sample of convenience. Every effort was made to obtain as large a sample size as possible with the resources available.

#### Setting

Participants were recruited from a community health centre in an area where the population (*n* = 391 749) is predominantly Black African and the majority come from low-income households (Statistics South Africa 2013). Those with PTB came from the HIV clinic at the health centre. Those without a history of PTB came from subjects in the main clinic who were not there to seek medical treatment, for example, in the company of family members seeking treatment. Recruiting participants from both the HIV and main clinics was done in order to create a mix of those with a current/past history of PTB and those without. Recruiting patients from the same area was done in order to control for social, cultural, ethical and economic factors.

## Inclusion and exclusion criteria

The inclusion and exclusion criteria were based on research done by Akkara *et al*. (2013), Louw, Goldin and Joubert (1996) and Brändli *et al*. (1996).

**Inclusion criteria:** Participants were included in the study if they presented with one or more of the following:

1. HIV positive and on HAART
2. Current PTB and on treatment
3. History of previous PTB and completed treatment
  OR
  If they were healthy, self-reported HIV-negative adults with no history of PTB.

Participants between the ages of 18 and 65 were selected. This age bracket was chosen as PTB has been shown to be prevalent in a working adult population (WHO 2014).

In order to ensure all participants were similar in the areas of age, race, socio-economic status (SES) and sex, all subjects were recruited from the same geographical area where most of the population come from a similar socio-economic background.

**Exclusion criteria:** The exclusion criteria were as follows:

1. History of severe chest infection, bronchitis or pneumonia
2. History of any severe chest trauma
3. Current history of asthma, chronic bronchitis, emphysema, bronchiectasis, cardiac failure or other diseases that affect the respiratory system
4. History of occupation that involves working on mines or building sites or exposure to dust, chemical fumes or gas.

Additionally, participants with no current/past history of PTB were excluded if they were suffering from a cough and/or sputum production for more than 3 months of the year, wheeze or shortness of breath at rest or at night or any symptoms of TB.

## Procedure

Participants were approached by the researchers in the waiting rooms and invited to participate in the study. Those who agreed completed informed consent and a demographic questionnaire, after which they underwent anthropometric measurements and spirometry testing with the Medical International Research Spirolab III desktop spirometer – following the procedure outlined by Miller, Hankinson and Brusasco (2005).

No blinding of investigators was done as staff limitations required both the recruitment and testing to be carried out by the same researcher. This could lead to bias in the study.

## Ethical considerations

Ethical approval was obtained from the Human Research Ethics Committee of the University of Cape Town (Ref no. 101/2014) and the Western Cape Department of Health.

**Potential hazards and benefits:** Participants were warned that they may experience dizziness/shortness of breath after spirometry and were instructed on what to do if symptoms were experienced.

**Recruitment procedures:** Participants were addressed in the waiting room and invited to take part in the study. They were then taken to a consulting room by the researcher where the data collection procedure was explained and conducted. Participation was voluntary and consent could be withdrawn at any time without threat of negative consequences.

**Informed consent:** All participants received an information sheet and completed an informed consent form after the procedure was explained to them and before being included in the study.

**Data protection:** All data were stored in password-protected folders and participants were kept anonymous as all subjects were numbered with no information linking the participant name to their number.

## Statistical analysis and data management

All the data were managed using a Microsoft Excel spreadsheet and analysed using Statistica (STATISTICA 2014). Shapiro–Wilk tests were done on all variables to determine normality. Multiple regression analyses were performed with each lung function variable, that is, FEV_1_, FVC and FEV_1_:FVC, being the dependent variables. Age, sex, current smoking, body mass index, current PTB, past PTB and HIV status were used as the independent variables. Dummy variables were used for all categorical data. Forward stepwise regression was used and residual analysis was done. Outliers were then removed and the final models presented.

Pearson chi-squared analysis and Fisher exact tests were performed to determine if there was an association between PTB (past/current) and the presence of COPD according to the GOLD standards of classification which classifies FEV_1_:FVC < 70% predicted and FEV_1_ < 80% predicted as indicating obstruction (Dewar & Curry 2006).

## Results

Sixty-three participants underwent testing. Eight were excluded as they were unable to follow the instructions and perform the lung function test effectively as outlined in the acceptability criteria of Miller, Hankinson and Brusasco (2005). In total, 55 subjects between the ages of 21 and 65 were included in the study. The number of participants with each interested variable and other characteristics of the participants can be seen in Table 1.

**TABLE 1:** Descriptive statistics of sample (*n* = 55).

Variable	Number	Percentage	Mean*	Median	SD	Range
Male	15	27.27%	-	-	-	-
Female	40	72.73%	-	-	-	-
Employed	17	30.91%	-	-	-	-
HIV positive	43	78.18%	-	-	-	-
Currently smoking	12	21.82%	-	-	-	-
Current PTB	12	21.82%	-	-	-	-
Previous PTB	15	27.27%	-	-	-	-
No PTB	12	21.82%	-	-	-	-
Mass (in kg)	-	-	73.7	-	15.99	-
Height (in cm)	-	-	162.55	-	7.93	-
Age (in years)	-	-	-	38	-	21–65
BMI	-	-	-	26	-	17.08–60.23
Income per person in household (in ZAR)	-	-	-	R288.89	-	R0–3600

*Source*: Authors’ own work

PTB, pulmonary tuberculosis; *, Normally distributed data represented as mean and SD Non-parametric data represented as median and range.

After residual analysis, four outliers were removed from the final model predicting FEV1. Current PTB reduced FEV1 by 23.39% (*p* < 0.001) and previous TB reduced it by 11.76% (*p* = 0.027). The adjusted *R*^2^ was 0.24, which indicated that the model accounted for 24% of the variance. (See Table 2.)

**TABLE 2:** FEV1 % predicted regressional analysis with 4 outliers removed and forward stepwise analysis.

*N = 51*	*β*	SE of *β*	*b*	SE of *b*	*t (48)*	*p-value*
Intercept	-	-	94.19	3.008	31.31	0
Current TB: no = 0, yes = 1	-0.524	0.13	-23.39	5.786	-4.04	0.0002
Previous TB: no = 0, yes = 1	-0.296	0.13	-11.76	5.148	-2.28	0.027

*Source*: Authors’ own work

Regression summary for dependent variable: FEV1 % Pred. *R* = 0.51756752; *R*² = 0.26787614; Adjusted *R*² = 0.23737097.

*F* = 8.7813; *df* = 2.48; *p* < 0.00056; SE: 15.631.

Three outliers were removed in the model predicting FVC. Current PTB and previous PTB both reduced FVC, with current PTB reducing it by 15.99% (*p* < 0.001)) and previous PTB by 10.79% (*p* = 0.031). The model was weaker than that predicting FEV1 with an adjusted *R*^2^ = 0.20. (See Table 3.)

**TABLE 3:** FVC % predicted regressional analysis with 3 outliers removed and forward stepwise analysis.

*N = 52*	*β*	SE of *β*	*b*	SE of *b*	*t (48)*	*p- value*
Intercept	-	-	90.54	4412	20.52	0
Current TB: no = 0, yes = 1	-0.41	0.134	-15.99	5211	-3.07	0.004
Previous TB: no = 0, yes = 1	-0.293	0.132	-10.79	4853	-2.22	0.031
Sex: m = 0, f = 1	0.213	0.129	7.66	4631	1.65	0.104

*Source*: Authors’ own work

Regression summary for dependent variable: FVC % Pred. *R* = 0.49652264; *R*² = 0.24653473; Adjusted *R*² = 0.19944316.

*F* = 5.2352; *df* = 3.48; *p* < 0.00331; SE of estimate: 14.399.

Three outliers were removed from the forward stepwise regression model predicting FEV1:FVC. The variables that remained included age, which reduced FEV1:FVC by 0.26% per year (*p* = 0.015); current PTB, which reduced it by 6.44% (*p* = 0.017); and previous PTB, which reduced it by 4.88% with a *p*-value approaching significance (*p* = 0.059). The adjusted *R*2 value was 0.18. (See Table 4.)

**TABLE 4:** FEV1:FVC regressional analysis with 3 outliers removed and forward stepwise analysis.

*N = 52*	*β*	SE of *β*	*b*	SE of *b*	*t (48)*	*p-value*
Intercept	-	-	93.77	4.403	21.3	0
Age	-0.323	0.128	-0.26	0.104	-2.53	0.015
Current TB: no = 0, yes = 1	-0.332	0.134	-6.44	2.598	-2.48	0.017
Previous TB: no = 0, yes = 1	-0.259	0.134	-4.88	2.528	-1.93	0.059

*Source*: Authors’ own work

Regression summary for dependent variable: FEV1:FVC *R* = 0.47374617; *R*² = 0.22443544; Adjusted *R*² = 0.17596265.

*F* = 4.6301; *df* = 3.48; *p* < 0.00635; SE of estimate: 7.4795.

Six subjects (10.9%) were classified as having COPD according to the GOLD standard (Dewar & Curry 2006). It was found that half of these subjects (5.45%) had previous PTB. A chi-squared test to determine whether there was an association between COPD and past TB did not yield significant results with a chi-square of 1.75 (*p* = 0.19) and a Fisher’s exact *p*-value of 0.196.

## Discussion

### Outline of results

This study found that lung function was significantly reduced in subjects with current or past PTB, but did not find any association between PTB and COPD.

All lung parameters (FEV1, FVC and FEV1:FVC) were significantly decreased in subjects with current PTB. This finding is in keeping with the literature (Long *et al*. 1998; Maguire *et al*. 2009) and may be directly due to the effects of the mycobacteria on the lungs, namely cavitation, loss of lung parenchyma, loss of volume and calcification (American Thoracic Society 2000) as structural changes have a direct effect on lung function (Long *et al*. 1998).

Both FEV1 and FVC were significantly reduced in subjects with a past history of PTB. FEV1:FVC was also reduced, although not significant (*p* = 0.059); the only value in this study which does not support the literature. In a larger study by Akkara *et al*. (2013), the authors found that, of 257 participants with cured PTB, 86.8% had spirometry readings consistent with obstructive airway disease, that is, FEV1:FVC < 70%. Ehrlich *et al*. (2011a) echo this finding in their review of South African literature that showed a significant association between chronic airway obstruction and PTB; however, many of these studies were conducted on miners with very few population-based studies being included.

Although six subjects (10.9%) were classified as having COPD, no association was found between past PTB and COPD. This is again in contrast to the literature (Allwood *et al*. 2013; Baig, Saeed & Khalil 2010; Ehrlich *et al*. 2011a) and may be due to the small sample size of the study, which could in turn influence the power of the study.

Hnizdo *et al*. (2000) suggest that the decrease in lung function may be due to residual damage to lung tissue after initial infection and completion of TB treatment. In three large population studies, the authors found a significant association between history of PTB and presence of airflow obstruction according to spirometry (Caballero *et al*. 2008; Lam *et al*. 2010; Menezes *et al*. 2007). These findings demonstrate PTB as an important cause of chronic lung disease, which can lead to significant disability (Pasipanodya *et al*. 2007).

FEV1:FVC was also found to decrease with advancing age. This is unsurprising as the structural and physiological changes, and resultant decline in lung function that comes with increased age, are well established in the literature (Sharma & Goodwin 2006; Wahba 1983).

HIV status did not seem to have a significant effect on lung function, which may suggest that the TB bacteria are the main cause for the reduced lung function and not the HIV. Although Kristoffersen *et al*. (2012) suggest that the virus itself may contribute towards decreased lung function by altering the thickness of alveolar membranes even in those without PTB. These authors, in their prospective study, found that diffusing capacity of the lungs for carbon monoxide (DLCO) tended to decline over time in those with HIV. However, they could not conclude whether this was due to the ARVs or the virus itself. More studies are needed to determine the exact contribution of HIV to lung impairment both in subjects with PTB and those without. This was beyond the scope of this study.

### Practical implications

This study’s findings have important implications for pulmonary rehabilitation and would suggest that patients who have undergone treatment for PTB may have need for further intervention in order to address residual impairments in lung function and exercise tolerance (Maguire *et al*. 2009). Physiotherapists, with their skills and knowledge regarding pulmonary rehabilitation, are in a unique position to address this need.

Traditionally, physiotherapists do not treat patients with PTB unless they need to assist in the mobilisation of secretions (Goodman & Fuller 2009). Hall and de Charmoy (2002), in their study of young male subjects with PTB, found no reductions in exercise capacity and suggested that the physiotherapist may play more of an educational role in PTB. The study, however, had a small sample size and very particular patient demographics, that is, young male subjects who may already be fitter than the general population by virtue of their gender and age. This may have significantly affected the generalisability of the results. A larger study by Ando *et al*. (2003) involving participants with cured PTB showed significant improvements in dyspnoea grade, activity score and 6-minute walking distance after a 9-week outpatient pulmonary rehabilitation programme. This lends credence to the authors’ opinion that health professionals, specifically physiotherapists, should play more of an active role in rehabilitation after PTB.

### Limitations of study

This study has a number of limitations, most notable being its small sample size, which significantly reduced the power of the study. Additionally, those with previous PTB were not homogenous in their PTB history with some subjects having had multiple episodes. This may impact on the degree of lung function impairment (Hnizdo *et al*. 2000). Those with current PTB were also measured at any stage of their treatment, which is important as research has shown differences in lung function at different stages of treatment (Maguire *et al*. 2009). The HIV-negative status of subjects with no PTB was not medically confirmed, which introduces another limitation if participants were not truthful or perhaps not aware that they were HIV positive. We also cannot conclude whether lung function deficits persist as participants were not followed up. Benito *et al*. (2012) have suggested that pulmonary complications occurring in HIV positive individuals are directly proportional to the decrease in CD4 lymphocytes. As CD4 counts were not measured, the authors were unable to determine whether lung function differs with CD4 count.

### Recommendations

Larger, population studies based in South Africa are needed to either confirm or refute these findings, and longitudinal studies are needed in order to determine whether lung function deficits persist long term and to what degree. In a country as severely affected by TB as South Africa is, serious consideration needs to be given to the need for programmes addressing functional lung impairments after patients have completed treatment. Further research regarding the types of interventions that can best address the functional lung impairments after PTB cure may be beneficial.

## Conclusion

Lung function was reduced both during and after treatment for PTB. These deficits may persist and present an important cause for chronic lung disease and disability. HIV did not significantly affect lung function. More research is needed to determine the contribution of HIV towards lung impairment in PTB. The power of this study was reduced because of its small sample size. However, the results still have important implications regarding the need for further intervention, that is, pulmonary rehabilitation, even after medical cure of PTB, in order to address residual impairments in lung function.
